# Impact of ovarian preservation on survival for premenopausal women with early‐stage endometrial carcinoma: A systematic review and meta‐analysis

**DOI:** 10.1002/ijgo.71101

**Published:** 2026-06-11

**Authors:** Antonio Raffone, Diego Raimondo, Daniele Neola, Manuela Maletta, Marisol Doglioli, Antonio Travaglino, Carlo Ronsini, Maria Giovanna Vastarella, Stefano Ferla, Francesco Cosentino, Pasquale De Franciscis, Renato Seracchioli, Luigi Cobellis

**Affiliations:** ^1^ Department of Woman, Child, and General and Specialized Surgery University of Campania “Luigi Vanvitelli” Naples Italy; ^2^ Department of Medical and Surgical Sciences (DIMEC) University of Bologna Bologna Italy; ^3^ Division of Gynaecology and Human Reproduction Physiopathology IRCCS Azienda Ospedaliero‐Universitaria di Bologna Bologna Italy; ^4^ Department of Neuroscience, Reproductive Sciences and Dentistry, School of Medicine University of Naples Federico II Naples Italy; ^5^ Unit of Pathology, Department of Medicine and Technological Innovation University of Insubria Varese Italy; ^6^ Gynecologic Oncology and Surgery Unit Responsible Research Hospital Campobasso Italy; ^7^ Department of Medicine and Health Science “V. Tiberio” University of Molise Campobasso Italy

**Keywords:** cancer, cardiovascular risk, endometrium, health, non‐alcoholic fatty liver disease, osteoporosis, uterus

## Abstract

**Objective:**

Up to 14% of women with early‐stage endometrial carcinoma (EEC) are diagnosed before menopause. Although ovarian preservation (OP) within surgical staging might avoid surgical menopause, its rate remains low in clinical practice, probably because the impact of OP on survival outcomes is unclear. This study evaluates the impact of OP on (i) survival outcomes, (ii) adverse events, and (iii) quality of life (QoL) in young women with EEC.

**Method:**

A systematic review and meta‐analysis was performed searching seven electronic databases from their inception to 2022. The study included all randomized controlled trials (RCTs) and non‐randomized comparative studies (NRS) comparing survival outcomes of women who underwent OP versus bilateral salpingo‐oophorectomy (BSO). Primary outcome was the risk of death of any cause, while secondary outcomes were the risk of death due to endometrial carcinoma, the risk of recurrent disease, adverse events after ovarian surgery, and QoL.

**Results:**

Twelve studies were included in the qualitative synthesis, while nine studies with a total of 115 528 women were suitable for the quantitative analysis. Compared to the BSO group, the OP group had a hazard ratio of 0.805 (95% confidence interval [CI]: 0.655–0.989) for death of any cause, 1.079 (95% CI: 0.395–2.953) for death due to endometrial carcinoma, and 1.332 (95% CI: 0.663–2.674) for recurrent disease. No pooled data were obtainable for adverse events after ovarian surgery and QoL.

**Conclusion:**

In young women with EEC, our study seems to support the OP at the time of surgical treatment as it appears to reduce the risk of death of any cause by approximately 20%, with no impact on the risk of death due to endometrial carcinoma and the risk of recurrent disease. However, given the risk of bias of the included studies, further well‐designed studies are needed.

## 1 INTRODUCTION

Endometrial carcinoma is the most common gynecological malignancy in high‐income countries.^1^, ^2^, ^3^, ^4^ Known risk factors for endometrial carcinoma are high levels of estrogens without the protective effect of progesterone/progestins (unopposed estrogens), obesity, early age at menarche, nulliparity, late age at menopause, Lynch syndrome (i.e., a hereditary syndrome resulting from germline mutations in DNA mismatch repair genes, such as *MLH1*, *MSH2*, *MSH6*, and *PMS2*, and characterized by familial clustering of colorectal, endometrial, and other types of cancer^5^), older age, and tamoxifen use.^6^ Due to a high prevalence of such risk factors, the incidence of endometrial carcinoma has increased over the past two decades in the USA.^2^, ^7^

The standard surgical treatment of early‐stage endometrial carcinoma (EC) is total hysterectomy and bilateral salpingo‐oophorectomy (BSO) with sentinel lymph node biopsy^8^, ^9^, ^10^ Although endometrial carcinoma is more common in post‐menopausal women, up to 14% of cases are diagnosed before menopause,^11^ and 5% are diagnosed before age 40.^12^ In pre‐menopausal women, BSO at time of hysterectomy leads to a surgical menopause, which might be associated with more severe and prolonged menopausal symptoms,^9^, ^13^, ^14^ metabolic changes (e.g., changes in lipid profile and increased insulin resistance^15^), and increased risk of cardiovascular events,^16^ osteoporosis,^17^ non‐alcoholic fatty liver disease,^18^ cognitive decline and dementia.^19^

Therefore, pre‐menopausal women with EC might benefit from ovarian preservation (OP). According to the guidelines of the European Society of Gynecological Oncology,^20^ the British Gynecological Cancer Society,^21^ and the National Comprehensive Cancer Network,^22^ OP can be considered in pre‐menopausal women with early‐stage endometrial carcinoma (EEC), defined as a low‐grade endometrioid endometrial carcinoma, with less than 50% myometrial invasion and no macroscopic ovarian or other extra‐uterine disease. OP is not recommended in women with EC with a family history involving ovarian cancer risk (e.g., BRCA mutation, Lynch syndrome).^6^, ^23^

In women with EEC, OP consists of ovarian sparing during surgical treatment, which includes total hysterectomy with bilateral salpingectomy and lymph node staging.^6^, ^23^ The main risks of OP are synchronous ovarian cancer and occult ovarian endometrial carcinoma metastases (i.e., dissemination of cancer cells from the uterine primary lesion to ovaries) and impact of estrogens produced by the spared ovaries on endometrial carcinoma recurrence.^24^

Although OP can avoid short‐ and long‐term consequences of surgical menopause in young women undergoing surgical treatment for early‐stage, low‐grade, endometrioid endometrial carcinoma^9^ and is included in international guidelines,^6^, ^23^ the rate of OP remains low in clinical practice: fewer than 10% of eligible women.^9^ As a result, most young women with early‐stage, low‐grade, endometrioid endometrial carcinoma undergo BSO at the time of surgery.^24^ An explanation might be that the impact of OP on survival outcomes remains unclear. Two systematic reviews and meta‐analyses reported no significant difference in overall survival between women with EEC treated with OP and those treated with BSO (hazard ratio [HR]: 1.00, 95% confidence interval [CI]: 0.72 to 1.39, and HR: 0.65, 95% CI: 0.38 to 1.11, respectively).^25^, ^26^ In another systematic review and meta‐analysis, an improved overall survival was reported among women who had spared their ovaries, with a 25% decreased in risk of death of any cause (HR: 0.75, 95% CI: 0.57 to 0.99).^27^ However, disease‐free survival was only assessed in two out of three of the abovementioned systematic reviews and meta‐analysis.^25^, ^27^ In particular, such studies did not find a significant difference between women treated with OP and those treated with BSO (HR: 1.49, 95% CI: 0.56 to 3.93, and HR: 1.22, 95% CI: 0.32 to 4.72, respectively).^25^, ^27^

This systematic review aims to update and improve knowledge about the impact of OP on survival outcomes in EEC in young women, helping to clarify whether and when it might be possible to avoid the consequences of surgical menopause. In particular, our review aims to inform surgical decision‐making, and improve the understanding of the potential implications of OP for young women with EEC. The primary objective is to evaluate the impact of OP on survival outcomes of young women with EEC, while the secondary objectives are to evaluate the effect of OP on adverse events and quality of life (QoL).

## 2 MATERIALS AND METHODS

### 2.1 Study protocol

The study followed an a priori protocol that defined each review step and is registered and published by the Cochrane International database of Systematic Reviews.^28^ All review stages, including search strategy, study selection, risk of bias assessment, data extraction, and data analysis, were independently performed by two review authors (D.N and M.M). Disagreements were resolved by discussion with a senior author (A.R). The Cochrane Handbook for Systematic Reviews of Interventions was adopted for reporting of the whole study.^29^ The results of the searches and of the process of screening and selecting studies for inclusion in the review were graphically reported in a PRISMA flow chart.

### 2.2 Study outcomes

Primary outcome was the risk of death of any cause in the “OP group” of patients compared to the “BSO group” of patients during the time from surgery until all‐cause mortality.

Secondary outcomes were:

- the risk of death due to endometrial carcinoma in the OP group compared to the BSO group
- the risk of recurrent disease (based on either clinical evidence or imaging confirmation) in the OP group compared to the BSO group
- the risk of adverse events after ovarian surgery (e.g., severe and prolonged menopausal symptoms, metabolic changes, cardiovascular events, osteoporosis, non‐alcoholic fatty liver disease, dementia, cognitive decline, and synchronous ovarian cancer)^30^ in the OP group compared to the BSO group
- the difference in QoL: assessed through specific tools in the included studies (e.g., the Quality of Life Scale (QOLS))^31^; the 36‐item Short Form Health Survey (SF‐36)^32^; European Organization for Research and Treatment of Cancer Quality of Life Questionnaire (EORTC QLQ)–C30^33^, ^34^; QLQ Endometrial Cancer Module (QLQ‐EN24)^33^; Functional Assessment of Cancer Therapy‐General (FACT‐G).^34^

Primary and secondary outcomes were considered for building Cates plots.^35^

### 2.3 Search strategy

We searched the following databases for systematic reviews and studies and checked the reference lists of those that were relevant for additional studies:

- Cochrane Central Register of Controlled Trials (CENTRAL; latest issue), in the Cochrane Library;
- MEDLINE via Ovid (1946 to December 2022);
- Embase via Ovid (1980 to December 2022).

The MEDLINE search strategy is presented in Supporting Information S1. For databases other than MEDLINE (Ovid and CENTRAL), the search strategy was adopted accordingly (Supporting Information S2 and S3).

Other searching resources were: PubMed, Web of Sciences, Google Scholar, and ClinicalTrial.gov, from their inception to December 2022.

Language restrictions were not applied in order to reduce the language bias, according to Chapter 6 of *the Cochrane Handbook for Systematic Reviews of Interventions*.^36^

### 2.4 Study selection

We included all randomized controlled trials (RCTs) and non‐randomized comparative studies (NRS; i.e., cohort and case–control studies) comparing survival outcomes of women 18 years of age or older who underwent OP versus BSO at time of surgical treatment for endometrial carcinoma. Case reports and case series (*n* < 10) were a priori considered as exclusion criteria.

### 2.5 Data extraction

Data extraction was performed without modification of original data. We extracted data using a pre‐designed Excel file before reporting the data into RevMan Web.^37^ Data were extracted independently by all review authors working in pairs. Disagreements were resolved by discussion with a senior author. When information was unclear, we contacted authors of the original studies to clarify and provide further details.

### 2.6 Risk of bias within studies assessment

Two review authors (A.R and D.N) independently assessed the risk of bias of the included studies. Criteria outlined in the *Cochrane Handbook for Systematic Reviews of Interventions* ^29^ were used to assess the risk of bias within RCTs, while the Risk of Bias In Non‐randomized Studies‐of Interventions (ROBINS‐ I) tool^38^ was used for assessing risk of bias within NRS.

In particular, when using the ROBINS‐I tool, we assessed the risk of bias of studies based on the following seven domains:

- bias to due confounding
- bias in selection of participants into the study
- bias in classification of interventions
- bias due to deviations from the intended intervention
- bias due to missing data
- bias in measurement of outcomes
- bias in selection of reported results.

Our risk of bias assessment categorized the studies in five domains as having either a “critical risk,” “serious risk,” “moderate risk,” “low risk,” or “no information.” The criteria by which we determined the risk of bias evaluation are outlined in Table S1. If the two review authors disagreed, the matter was resolved through discussion or by consulting a third review author (R.S). As per the guidelines of the ROBINS‐I tool, we classified an NRS as low risk if all domains were judged to be at low risk of bias; as moderate risk if all domains were assessed as either low or moderate risk of bias; as serious risk if at least one domain had a serious risk of bias but none had a critical risk; and as critical risk if at least one domain was deemed to have a critical risk of bias. If there was insufficient information for any key domain, we labeled it as “no information on an NRS” unless there was clear evidence of serious or critical risk of bias. For NRS, we also reviewed whether covariance analyses were performed, as these analyses adjust the results for the potential influence of variables other than the intervention of interest, thereby reducing the risk of bias.

### 2.7 Data analysis

The hazard ratio (HR) for death of any cause, death due to endometrial carcinoma, and recurrent disease, the odds ratio (OR) for adverse events, and the mean difference in QoL scores were reported for each included study and as pooled estimate on forest plots, with 95% CI, for both univariable and multivariable analyses from included studies. BSO groups were considered as a reference. We adopted the random effect model of DerSimonian and Laird for all analyses.^39^

We estimated statistical heterogeneity among the included studies with the Higgins *I* ^2^‐statistic; heterogeneity was a priori classified as null for *I* ^2^ = 0%, minimal for 0% < *I* ^2^ ≤ 25%, low for 25 < *I* ^2^ ≤ 50%, moderate for 50 < *I* ^2^ ≤ 75%, and high for *I* ^2^ > 75%, as previously reported.^40^, ^41^, ^42^

We used Comprehensive Meta‐Analysis for statistical analysis of data.^43^

Additional analyses were performed as subgroup analyses based on factors able to impact prognosis on women with endometrial carcinoma, such as age (45 years or younger, 50 years or younger); severe comorbidity (based on a cut‐off of 5 using the Charlson Comorbidity Index)^44^; obesity; histotype (endometrioid, non‐endometrioid); FIGO grade (1–2, 3); lymph vascular space invasion (no LVSI, focal LVSI, substantial LVSI); 2014 FIGO stage (IA, IB, II‐IV); World Health Organization Performance Status (WHO PS, 0, 1 to 2, 3 to 5); adjuvant treatment; molecular prognostic group (*POLE*‐mutated, mismatch repair proteins deficiency, p53 abnormal expression, and not specific molecular profile).^1^

## 3 RESULTS

### 3.1 Study selection

At the end of the databases searches, 26 796 studies were identified. Duplicates removal processes led to 1743 studies. Abstract and title screening led to 24 studies that were evaluated for eligibility. Of them, 12 studies were excluded:

- two were ongoing trials with no data published up to date^45^, ^46^
- for one study, the difference in the risk of survival outcomes between the two groups of patients was not extractable^47^
- four studies did not compare survival outcomes between OP and non‐OP groups^48^, ^49^, ^50^, ^51^
- five studies comparison of survival outcomes between the two groups were exclusively reported as Kaplan–Meir curves and, thus, not suitable for the risk assessment.^52^, ^53^, ^54^, ^55^, ^56^

Finally, 12 studies were included in qualitative synthesis^8^, ^9^, ^26^, ^57^, ^58^, ^59^, ^60^, ^61^, ^62^, ^63^, ^64^, ^65^ and nine studies were suitable for quantitative analysis,^58^, ^60^, ^61^, ^62^, ^63^, ^65^, ^66^, ^67^, ^68^ because three studies were judged at critical risk of bias^26^, ^58^, ^61^ (Figure S1).

### 3.2 Studies and patients' characteristics

We extracted data for a total of 119 629 women (7347 women in the OP group and 112 282 women in the BSO group) from 12 studies. The included studies were all observational retrospective cohort studies.^8^, ^9^, ^26^, ^57^, ^58^, ^59^, ^60^, ^61^, ^62^, ^63^, ^64^, ^65^

Six studies were National register‐based studies. In particular, in five studies^9^, ^58^, ^59^, ^63^, ^64^ authors analyzed data from the United States Surveillance, Epidemiology, and End Results (SEER) registers, while in one study^61^ authors analyzed data extracted from the National Cancer Database of the United States. The characteristics of the included studies are reported in Table 1.

**TABLE 1:** Characteristics of the included studies.

First author/year	Country	Population of interest, *n*	OP, *n* (%)	BSO, *n* (%)	Study design	Patients selection criteria	Outcomes	Period of enrollment
Wright 2009	United States^a^	3269	402 (12)	2867 (88)	National register retrospective cohort	Premenopausal women (≤45 years) with stage I endometrioid endometrial adenocarcinoma of any grade	OS and DSS	1988–2004
Lee 2013	Republic of Korea	495	176 (36)	319 (64)	Multi‐center retrospective cohort	Premenopausal women with stage I or II endometrioid endometrial adenocarcinoma of any grade	DFS and OS	1997–2008
Sun 2013	China	166^b^	34 (20.5)	132 (79.5)	Single‐center retrospective cohort	Premenopausal women (≤45 years) with early‐stage endometrial adenocarcinoma of any grade	OS	2000–2010
Lau 2014	Taiwan	529	17 (3)	512 (97)	Single‐center retrospective cohort	Women with endometrial adenocarcinoma of any grade	DFS	2000–2010
Gonthier 2016	France^a^	994	145 (15)	849 (85)	National register retrospective cohort	Premenopausal women (≤45 years) with stage I endometrial adenocarcinoma of grade 2 or 3	OS and DSS	1983–2012
Matsuo 2016	United States^a^	86 005	4109 (5)	81 896 (95)	National register retrospective cohort	Women with stage I endometrioid endometrial adenocarcinoma of grade 1 or 2	OS and DSS	1983–2012
Wright 2016	United States^a^	15 648	1121 (7)	14 527 (93)	National register retrospective cohort	Premenopausal women (≤50 years) with stage I endometrioid endometrial adenocarcinoma of any grade	OS	1998–2012
Hou 2017	China^a^	7183	863 (12)	6320 (88)	National register retrospective cohort	Premenopausal women (≤50 years) with stage I endometrioid endometrial adenocarcinoma of any grade	OS and DSS	2004–2013
Matsuo 2019	United States and Japan	1691	156 (9)	1535 (91)	Multicenter retrospective cohort	Premenopausal women (≤50 years) with stage I endometrioid endometrial adenocarcinoma of grade 1 or 2	OS and DFS	2000–2014
Shin 2020	Republic of Korea	539	70 (13)	469 (87)	Single‐center retrospective cohort	Women with stage I or II endometrioid endometrial adenocarcinoma of any grade	OS and DFS	2006–2017
Akgor 2022	Turkey, Belarus, Greece, Russia, Kazakhstan, Armenia, Azerbaijan	169	54 (32)	115 (68)	Multicenter retrospective cohort	Premenopausal women (≤40 years) with stage I endometrial adenocarcinoma of any grade	OS and DFS	2007–2019
Nasioudis 2022	United States	2941	200 (7)	2741 (93)	National register retrospective cohort	Premenopausal women (≤45 years) with stage I endometrioid endometrial adenocarcinoma of grade 2 or 3	OS	2004–2015
Total	‐	119 629	7347 (6)	112 282 (94)	‐	‐	‐	‐

Abbreviations: BSO, bilateral salpingo‐oophorectomy; DFS, disease‐free survival; DSS, disease‐specific survival; OP, ovarian preservation; OS, overall survival.

^a^ Authors analyzed data from the United States Surveillance, Epidemiology, and End Results (SEER) registers.

^b^ The study included 203 patients, however, all the patients with ovarian preservation were early stage; thus, only 166 patients with early‐stage endometrial cancer were analyzed to avoid bias.

The age of women with EEC treated with total hysterectomy ±BSO ranged from to 30 to 74 years. In all included studies, BSO was offered to women with EEC, while only two studies^57^, ^61^ reported data about OP in women with non‐endometrioid EEC. All the studies included women with stage IA or IB endometrial carcinoma according to FIGO 2009.^66^ Only one study^62^ also included stage II endometrial carcinoma women. The characteristics of patients in the included studies are reported in Table 2.

**TABLE 2:** Characteristics of patients of the included studies.

First author/year	Age, mean ± SD or range [years]			Histotype				FIGO grade				FIGO stage						Adjuvant treatment		Death of any cause		Death due to EC		Recurrence		Follow‐up Time, mean ± SD or median (range) [months]
	Population	OP	BSO	Endometrioid		Non‐Endometrioid		1 or 2		3		IA		IB		II		OP	BSO	OP	BSO	OP	BSO	OP	BSO	
				OP	BSO	OP	BSO	OP	BSO	OP	BSO	OP	BSO	OP	BSO	OP	BSO									
Wright 2009	30–45	NR	NR	NR	NR	NR	NR	376	2537	11	185	390	2736	12	131	0	0	10	243	NR^a^	NR^a^	NR^a^	NR^a^	NR	NR	NR
Lee 2013	35–41	NR	NR	176	319	0	0	173	303	3	16	159	283	6	17	0	0	19	54	NR^a^	NR^a^	NR^a^	NR^a^	4	8	49.0 (6–208)
Sun 2013	40 ± 4.7	NR	NR	34	127	0	5	12	37	22	95	29	126	5	6	0	0	6	28	NR^a^	NR^a^	NR	NR	NR	NR	120
Lau 2014	55 ± 11.4	41.8 ± 11.2	56.2 ± 11.2	17	457	0	55	16	430	1	81	NR						NR	NR	NR	NR	NR	NR	0	35	37.5 ± 30.1
Gonthier 2016	NR	38.3 ± 5.7	39.6 ± 4.8	96	849	0	0	86	760	10	89	96	849	0	0	0	0	NR	NR	1	33	2	12	NR	NR	96 (1–296)
Matsuo 2016	NR	58.4 ± 15	61.6 ± 11.6	4109	81 896	0	0	4109	81 896	0	0	3705	75 341	0	0	0	0	NR	NR	NR^a^	NR^a^	NR^a^	NR^a^	NR	NR	360
Wright 2016	30–49	NR	NR	1121	14 527	0	0	995	12 582	992	52	997	12 945	67	1105	0	0	NR	NR	18	405	NR	NR	NR	NR	61.9 (32.5–92)
Hou 2017	45	39.6 ± 6.9	44 ± 5.5	NR	NR	0	0	729	5164	19	338	833	5870	30	450	0	0	25	529	NR^a^	NR^a^	NR^a^	NR^a^	NR	NR	120
Matsuo 2019^b^																										
USA cohort	NR	37.9 ± 6.9	42.6 ± 5.6	113	1083	0	0	113	1083	0	0	NR						3	74	0	20	NR	NR	NR^a^	NR^a^	49.2
Japan cohort	NR	36.3 ± 6.4	43 ± 5.6	43	452	0	0	43	452	0	0	NR						2	37	0	8	NR	NR	NR^a^	NR^a^	61.2
Shin 2020	52.7 ± 10.4	40.9 ± 9.6	54.4 ± 9.4	66	395	4	74	62	372	5	47	60	330	8	118	2	21	10	149	NR^a^	NR^a^	NR	NR	NR^a^	NR^a^	85 (6–142)
Akgor 2022	NR	35.4 ± 4.6	36 ± 3.8	52	104	2	11	51	98	3	12	47	94	7	21	0	0	4	14	NR^a^	NR^a^	NR	NR	2	5	59 (4–187)
Nasioudis 2022	NR	37	41	200	2741	0	0	163	2299	37	442	173	2340	20	319	0	0	34	463	NR^a^	NR^a^	NR	NR	NR	NR	68.3
Total	\	\	\	6027	102 950	6	145	6928	108 013	1103	1357	6489	100 914	155	2167	2	21	113	1591	19	466	2	12	6	48	\

Abbreviations: BSO, bilateral salpingo‐oophorectomy; EC, endometrial cancer; NR, not reported; OP, ovarian preservation.

^a^ The study only reported HR for this outcome, not an absolute number of cases.

^b^ Matsuo 2019 study included two different cohorts, one from United States and one from Japan.

### 3.3 Risk of bias within studies assessment

Our assessment of risk of bias using the ROBINS‐I tool in the 12 included NRS is shown in Table S2 and Figure S2. Based on the criteria formulated in the Methods section, we labeled three studies as “critical risk” studies^26^, ^58^, ^61^ and nine studies as “moderate risk” studies.^58^, ^60^, ^61^, ^62^, ^63^, ^65^, ^66^, ^67^, ^68^

After the exclusion of “critical risk of bias” studies, we quantitatively analyzed data from nine studies^8^, ^9^, ^57^, ^59^, ^60^, ^62^, ^63^, ^64^, ^65^ for a total of 115 528 women (6968 in the OP group and 108 560 in the BSO group).

### 3.4 Meta‐analysis

#### 3.4.1 Primary outcome

Six studies reported HR for death of any cause and were included in this analysis. Compared to the BSO group, the OP group showed an HR of 0.805 (95% CI: 0.655 to 0.989; *I* ^2^ = 0%; Figure 1) for death of any cause.^9^, ^59^, ^62^, ^63^, ^64^, ^65^

**FIGURE 1:**
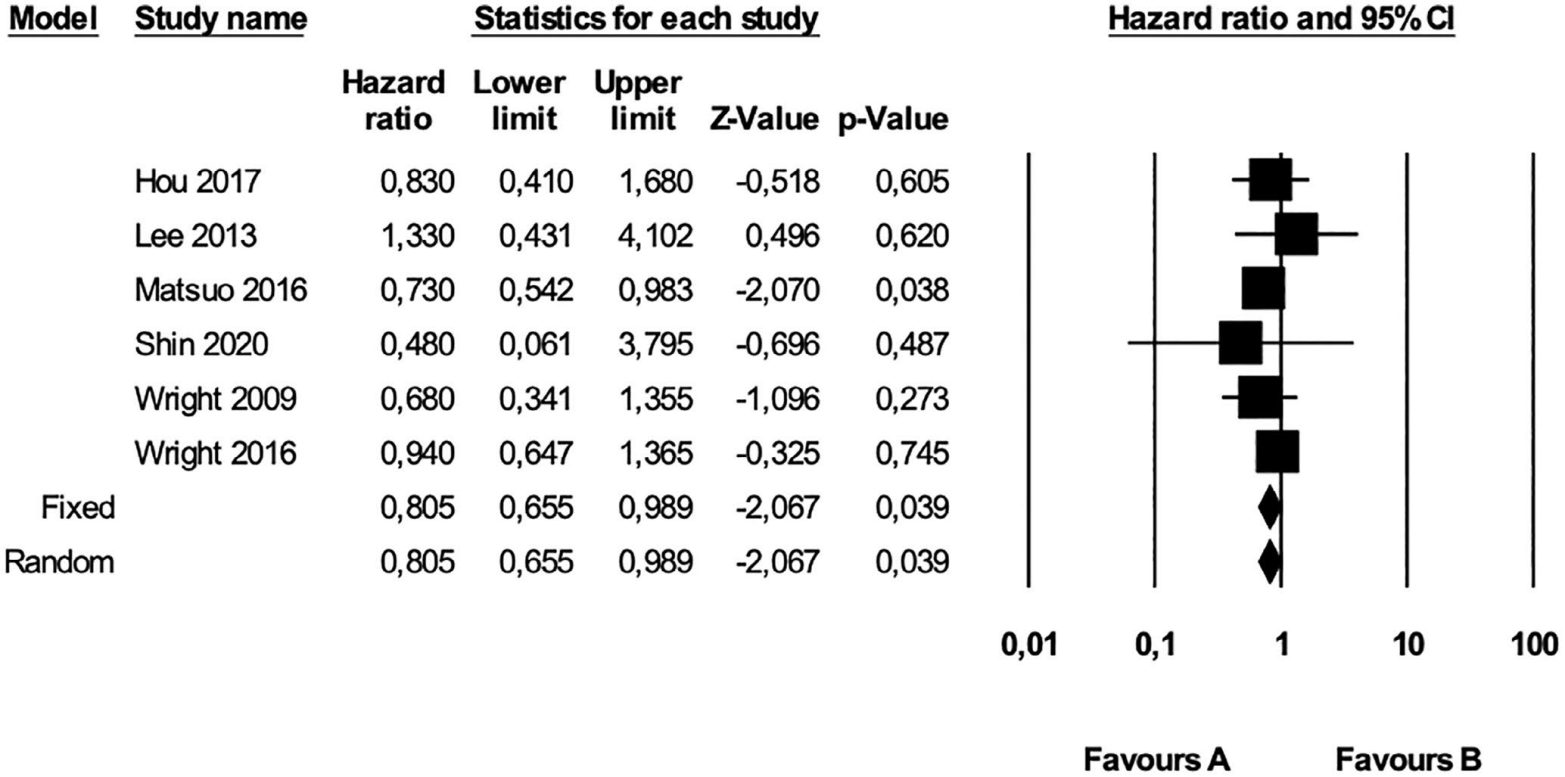
Forest plot of individual and pooled hazard ratio for death of any cause between ovarian preservation group and bilateral salpingo‐oophorectomy group of endometrial carcinoma (EEC) women; bilateral salpingo‐oophorectomy group was considered as a reference.

#### 3.4.2 Secondary outcomes

Two studies reported HR for death due to endometrial carcinoma and were included in this analysis. Compared to the BSO group, the OP group showed an HR of 1.079 (95% CI: 0.395 to 2.953; *I* ^2^ = 21.6%; Figure 2) for death due to endometrial carcinoma.^59^, ^63^

**FIGURE 2:**
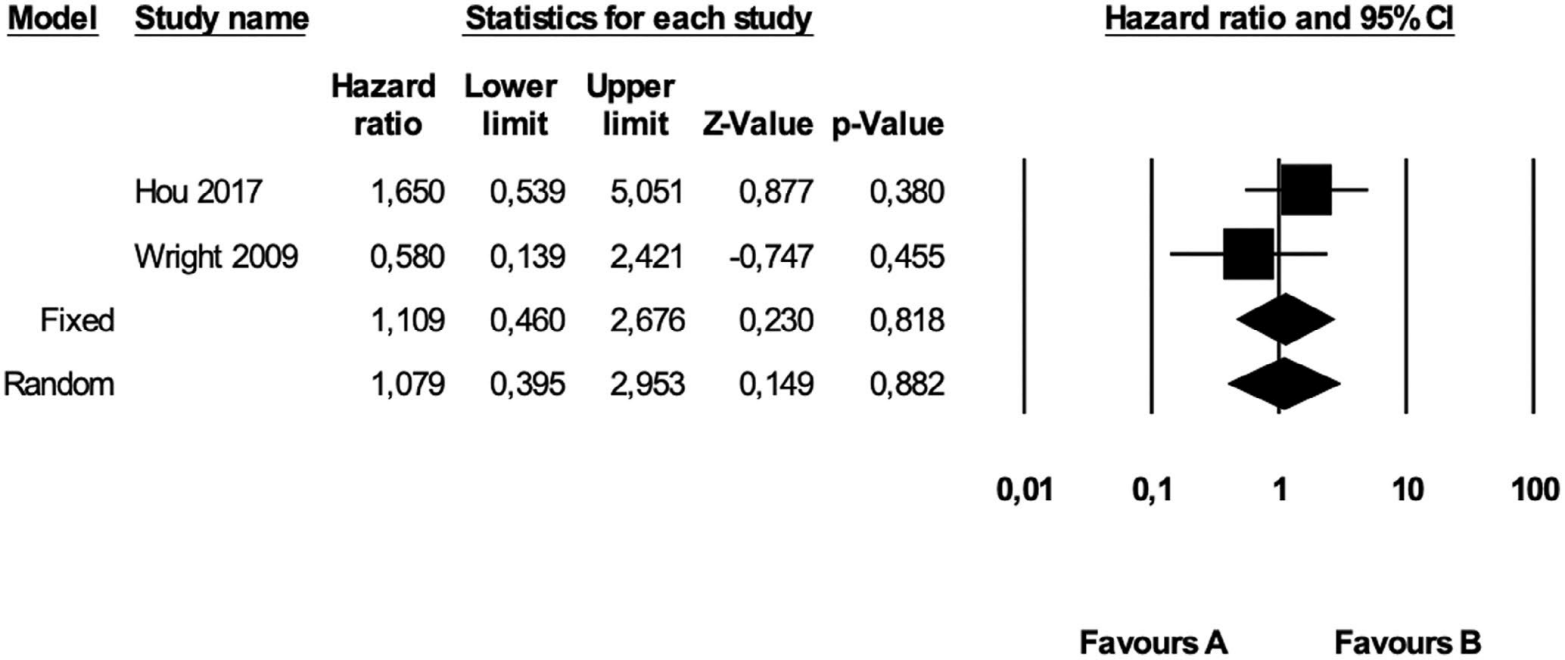
Forest plot of individual and pooled hazard ratio for death due to endometrial carcinoma between ovarian preservation group and bilateral salpingo‐oophorectomy group of endometrial carcinoma (EEC) women; bilateral salpingo‐oophorectomy group was considered as a reference.

Five studies reported HR for recurrent disease and were included in this analysis. Compared to BSO group, OP group showed an HR of 1.332 (95% CI: 0.663 to 2.674; *I* ^2^ = 40.4%; Figure 3) for recurrent disease.^9^, ^57^, ^60^, ^62^, ^65^

**FIGURE 3:**
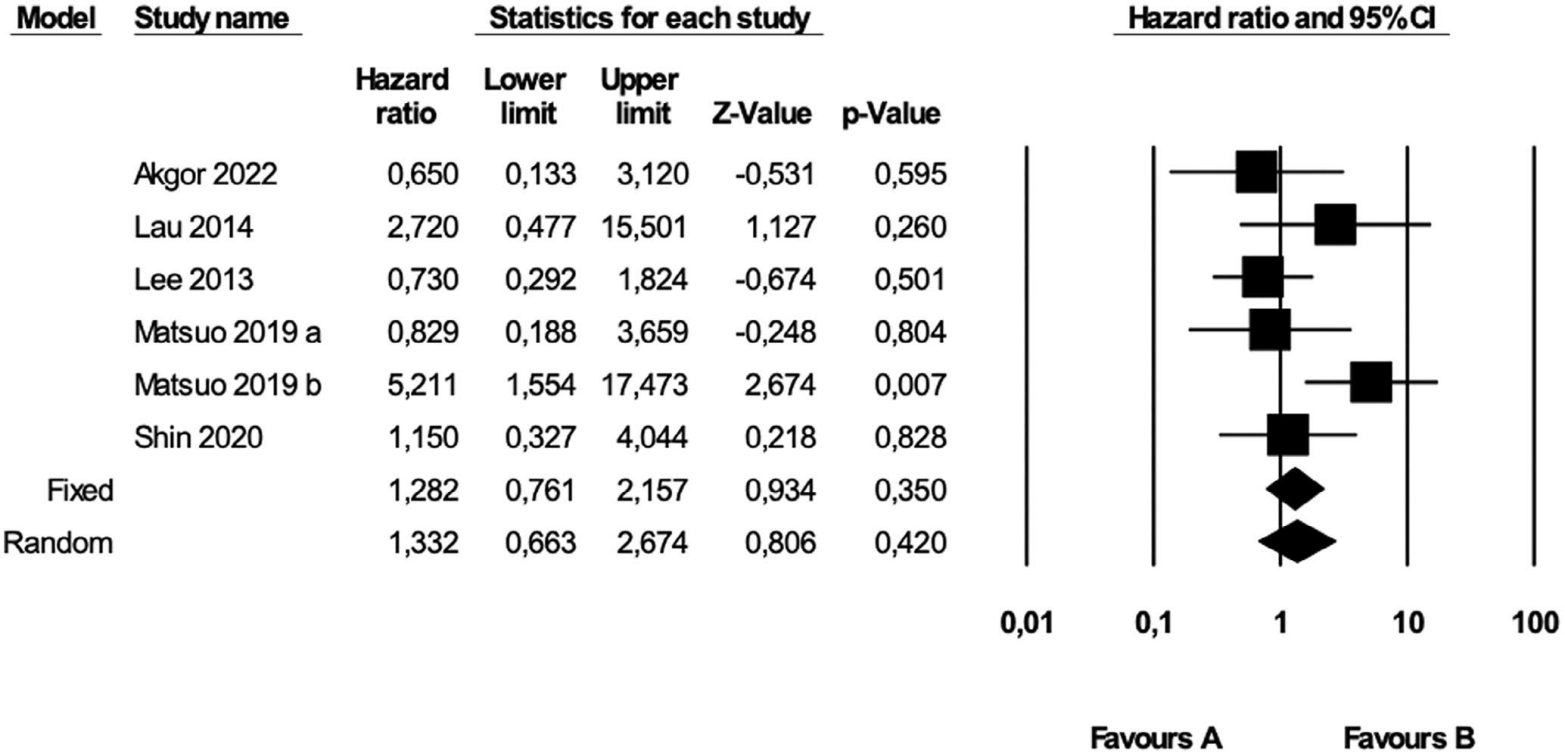
Forest plot of individual and pooled hazard ratio for recurrent disease between ovarian preservation group and bilateral salpingo‐oophorectomy group of endometrial carcinoma (EEC) women; bilateral salpingo‐oophorectomy group was considered as a reference.

Only one study assessed adverse events after ovarian surgery.^30^ This study reported an HR of 0.40 (95% CI: 0.17 to 0.91) for death from cardiovascular disease in women undergoing OP compared to women who had removed both ovaries.^9^

No included study assessed QoL; thus, a meta‐analysis was not feasible for this outcome.

#### 3.4.3 Subgroup analyses

Three studies were suitable for a subgroup analysis based on age ≤ 50 years. In women aged 50 years or younger, compared to the BSO group, the OP group showed an HR of 0.808 (95% CI: 0.648 to 1.008; *I* ^2^ = 0%; Figure S3) for death of any cause.^9^, ^59^, ^64^

Concerning the histotypes of endometrial carcinoma patients, four studies provided HR for death of any cause^9^, ^59^, ^64^, ^65^ and two studies^9^, ^65^ provided HR for recurrent disease in endometrioid histotype, respectively, thus were suitable for subgroup analyses. In women with endometroid histotype, compared to the BSO group, the OP group showed an HR of 0.823 (95% CI: 0.663 to 1.022, *I* ^2^ = 0%; Figure S4) for death of any cause and 1.453 (95% CI: 0.406 to 5.209, *I* ^2^ = 71.1%; Figure S5) for recurrent disease.

Regarding FIGO stage, four studies reported HR for death of any cause,^9^, ^59^, ^63^, ^64^ two studies reported HR death due to endometrial carcinoma,^59^, ^63^ and two other studies reported HR for recurrent disease in FIGO stage I,^8^, ^57^ respectively, thus were suitable for subgroup analyses. In women with FIGO stage I, compared to the BSO group, the OP group showed an HR of 0.795 (95% CI: 0.644 to 0.981, *I* ^2^ = 0%; Figure S6) for death of any cause, 1.079 (95% CI: 0.395 to 2.953, *I* ^2^ = 21.6%; Figure S7) for death due to endometrial carcinoma, and 1.520 (95% CI: 0.385 to 5.994, *I* ^2^ = 64.2%; Figure S8) for recurrent disease.

## 4 DISCUSSION

### 4.1 Main findings and interpretation

This review showed that in young women with EEC, OP significantly reduced the risk of death of any cause by approximately 20%, while it appeared to not affect the risk of death due to endometrial carcinoma and the risk of recurrent disease. These findings suggest that OP might be safe in young women with EEC, as it does not appear to worsen oncological outcomes.

Ovarian preservation in young women with EEC is an issue of increasing interest given the rising incidence of EEC in premenopausal women.^11^ In fact, OP avoids surgical menopause, which negatively affects women's survival and QoL. Premature (<40 years) and early menopause (between 40 and 45 years), either natural or surgical, have been reported as risk factors of cardiovascular disease events and cardiovascular mortality.^67^, ^68^, ^69^ The reduced risk of all‐cause death observed among women who preserved their ovaries might be linked to the protective effects of endogenous estrogens on cardiovascular health. Indeed, the sudden loss of estrogen can lead to vascular dysfunction, increased inflammatory cytokines, vascular damage, metabolic changes (i.e., higher lipid levels).^26^, ^69^ Further, OP does not appear to affect the risk of endometrial carcinoma‐related death or recurrence, supporting the low likelihood of occult ovarian metastases in early‐stage cases with normal‐appearing ovaries.^26^, ^49^, ^70^

The lack of impact of OP on the risk of death of any cause, in the subgroup analysis exclusively including women aged ≤50 years, might be explained by the reduced sample size compared to the main analysis, as this stratification was available in only three included studies.^9^, ^59^, ^64^ In fact, the difference appeared slightly not significant (HR of 0.808; 95% CI: 0.648 to 1.008).

### 4.2 Comparison with existing literature

Three systematic reviews and meta‐analyses evaluated survival outcomes of women with EEC who preserved their ovaries.^25^, ^26^, ^27^ Our findings confirm that OP significantly reduces the risk of death of any cause, aligning with Jia and Zhang.^27^ However, Gu et al. (2017) and Sun et al. (2013) found no significant difference in the risk of death of any cause.^25^, ^26^ The lack of impact observed by Gu et al. (2017)^25^ and Sun 2013^26^ might be due to the higher prevalence of women aged 45–50 years in the study populations, given that surgically induced menopause increases the risk of death of any cause in women under 45.^69^

Regarding disease recurrence, our findings agree with Gu et al. (2017)^25^ and Jia and Zhang (2017),^27^ showing no significant difference between women who preserved their ovaries and those who underwent BSO, confirming the low risk of occult ovarian metastases in early‐stage cases with normal‐appearing ovaries at hysterectomy.

### 4.3 Strengths and limitations

Our findings appear supported by good overall quality of the included studies, as revealed by the risk of bias within studies assessment: in fact, only three included studies were considered at critical risk of bias overall and were subsequently excluded through quantitative analysis.

Nevertheless, our study might have some limitations. First, we exclusively included studies with a retrospective observational design because of the absence of RCT available in the field. However, a risk of bias assessment according to the ROBINS‐I tool was performed and studies at critical risk of bias were excluded to reduce the weight on the findings. Second, the impossibility of evaluating the impact of OP on survival outcomes based on some conventional risk factors and molecular signature might limit the applicability of our findings. Indeed, in the last 10 years, the risk assessment of patients with endometrial cancer has integrated conventional and molecular risk factors using The Cancer genome ATLAS research Network (TCGA) and Proactive Molecular Risk Classifier for Endometrial Cancer (ProMisE).^71^, ^72^, ^73^ In fact, data about conventional and molecular risk factors were partially or totally lacking in the included studies, as well as data from multivariable analyses, with subsequent impossibility of assessing the prognostic role of OP independently from other prognostic factors.

Another potential limitation is the risk of duplication bias for the four studies with patients extracted from the SEER register.^9^, ^59^, ^63^, ^64^ However, this risk appears to be limited, as the periods of data collection reported in these studies had minimal overlap.

Finally, we were unable to evaluate adverse events related to ovarian surgery or compare QoL between the study groups as none of the included studies reported these outcomes. This limitation might affect the generalizability of our findings and their broader applicability in clinical practice.

## 5 CONCLUSION

Ovarian preservation during surgical treatment for young women with EEC seems to reduce the risk of death of any cause by approximately 20% without increasing the risk of death due to endometrial carcinoma or disease recurrence, particularly in patients with endometrial carcinoma at FIGO stage I.

Conclusions about the impact of OP on adverse events after ovarian surgery and QoL in these women could not be drawn because of the insufficient number of included studies to generate pooled data about such outcomes.

Future studies are necessary to confirm these findings and assess the prognostic independence of OP from other variables possibly affecting the prognosis. In addition, future studies should address adverse events after ovarian surgery as well as QoL.

## AUTHOR CONTRIBUTIONS

AR, DR, MM, and DN independently assessed electronic search, eligibility of the studies, inclusion criteria, risk of bias, data extraction and data analysis. DN and AR contributed to the elaboration of methods for risk of bias assessment, data extraction and analysis. AR, DR, AT conceived the study; MGV and FC worked on the design of the study; MD and SF worked on the manuscript preparation; FC, PDF, RS and LC supervised the whole study.

## FUNDING INFORMATION

The authors have nothing to report.

## CONFLICT OF INTEREST STATEMENT

Authors report no conflict of interest.

## Supporting information


**Data S1.** MOOSE checklist for meta‐analyses of observational studies.

**Data S2.** PRISMA_2020_checklist‐2.

**Figure S1.** Flowchart of study selection step of the systematic review and meta‐analysis (Prisma template [Preferred Reporting Item for Systematic Reviews and Meta‐analyses]).

**Figure S2.** Risk of bias summary according to ROBINS‐I item: review of authors' judgments about each risk of bias item for each included NRS.

**Figure S3.** Forest plot of individual and pooled hazard ratio for death of any cause between ovarian preservation group and bilateral salpingo‐oophorectomy group of EEC women aged 50 years or younger; bilateral salpingo‐oophorectomy group was considered as a reference.

**Figure S4.** Forest plot of individual and pooled hazard ratio for death of any cause between ovarian preservation group and bilateral salpingo‐oophorectomy group of endometrioid EEC women; bilateral salpingo‐oophorectomy group was considered as a reference.

**Figure S5.** Forest plot of individual and pooled hazard ratio for recurrent disease between ovarian preservation group and bilateral salpingo‐oophorectomy group of endometrioid EEC women; bilateral salpingo‐oophorectomy group was considered as a reference.

**Figure S6.** Forest plot of individual and pooled hazard ratio for death of any cause between ovarian preservation group and bilateral salpingo‐oophorectomy group of FIGO stage I EEC women; bilateral salpingo‐oophorectomy group was considered as a reference.

**Figure S7.** Forest plot of individual and pooled hazard ratio for death due to endometrial carcinoma between ovarian preservation group and bilateral salpingo‐oophorectomy group of FIGO stage I EEC women; bilateral salpingo‐oophorectomy group was considered as a reference.

**Figure S8.** Forest plot of individual and pooled hazard ratio for recurrent disease between ovarian preservation group and bilateral salpingo‐oophorectomy group of FIGO stage I EEC women; bilateral salpingo‐oophorectomy group was considered as a reference.

**Supporting Information S1.** MEDLINE search strategy.

**Supporting Information S2.** Central search strategy.

**Supporting Information S3.** Embase (Ovid) search strategy.

**Table S1.** Criteria for risk of bias judgments according to ROBINS‐I tool.

**Table S2.** Risk of bias assessment for the included studies through ROBINS‐I tool.

## Data Availability

Data sharing is not applicable to this article as no new data were created or analyzed in this study.
