# Supplementary material for: Impact of ovarian preservation on survival for premenopausal women with early‐stage endometrial carcinoma: A systematic review and meta‐analysis
Source: Int J Gynaecol Obstet. 2026 Jun 11;175(2):608–18. doi: 10.1002/ijgo.71101 (PMC13674844; doi:10.1002/ijgo.71101)
Supplement: Supplementary file 1 [file IJGO-175-608-s002.docx]

**Supplementary Table 2.** Risk of bias assessment for the included studies through ROBINS-I tool.

| **Study** | **Bias due to confondine** | **Bias in selection of participants into the study** | **Bias in classification of interventions** | **Bias due to deviations from the intended intervention** | **Bias due to missing data** | **Bias in measurement of outcomes** | **Bias in selection of the reported result** | **Overall risk of bias** |
| --- | --- | --- | --- | --- | --- | --- | --- | --- |
| **Wright 2009** | **Moderate** | **Low** | **Low** | **Low** | **Moderate** | **Low** | **Low** | **Moderate** |
| **Rationale for judgement** | While most important confounders were measured and adjusted for, some residual confounding may still exist, particularly with regard to unmeasured factors like hormonal status or genetics. Also, the study does not account for potential lifestyle or socioeconomic factors. | The selection process seems appropriate for the target trial, and no indication of significant bias from participant selection was noted. | The classification of the primary intervention (ovarian preservation) was accurate, with only minor uncertainties around adjuvant treatments like radiation. | No evidence of deviation from intended interventions was reported. | The censoring of patients who were lost to follow-up and missing data on radiation therapy might introduce some bias. | Outcome measurement was likely reliable, based on death records, which are an objective outcome measure. | The reported results align with the stated objectives, and no obvious signs of selective reporting were found. | The study seems well-conducted overall, but there are moderate concerns around potential confounding due to unmeasured factors and some missing data. |
| **Lee 2013** | **Moderate** | **Low** | **Low** | **Low** | **Moderate** | **Low** | **Low** | **Moderate** |
| **Rationale for judgement** | The study used appropriate statistical methods to adjust for confounders, but the lack of certain unmeasured confounders (e.g., hormonal status or genetic factors) could lead to some residual bias. | Selection into the study appears appropriate, with no major concerns about bias in participant inclusion. | The interventions (ovarian preservation VS BSO) were clearly defined and categorized based on surgical reports. | No apparent deviations from intended interventions. | While there is no indication of widespread missing data, the lack of detailed reporting on missingness for certain variables could introduce some bias. | Outcomes were objectively measured using clinical records, minimizing the risk of bias. | There is no indication of selective reporting, and all key outcomes were appropriately reported. | Moderate concerns arise due to potential unmeasured confounders and the possibility of some missing data. Overall, the risk of bias is moderate, but the results are likely to be reliable given the efforts to adjust for confounders and the thorough statistical approach employed. |
| **Sun 2013** | **Critical** | **Serious** | **Low** | **Low** | **Moderate** | **Serious** | **Moderate** | **Critical** |
| **Rationale for judgement** | The study acknowledges that younger patients and those with lower-risk disease were more likely to refuse bilateral oophorectomy, which may confound survival outcomes. Although the study adjusted for variables like tumor stage, grade, and lymph node metastasis, potential unmeasured confounders such as comorbidities or socioeconomic factors could influence the decision to preserve ovaries. The decision to preserve the ovaries was also influenced by intraoperative findings, which could reflect differences in disease severity that were not fully accounted for. | Patients self-selecting into the ovarian preservation group based on their preferences could lead to systematic differences between groups, although the study does attempt to control for major clinical factors. | There is minimal risk of misclassification, as the classification of intervention (BSO or ovarian preservation) is clearly documented in the surgical records. | The study does not report any deviations from the surgical interventions performed, and there is no indication of systematic differences in post-operative care between groups. | The study does not provide detailed information about loss to follow-up, which introduces uncertainty regarding the completeness of survival data. | Follow-up information was inconsistently obtained (e.g., phone calls for some patients), which could introduce bias in outcome measurement if there were differences in follow-up intensity between groups. | There is no mention of a pre-specified study protocol or planned analyses, which raises the possibility of selective reporting. | Potential confounding by patient choice, and incomplete follow-up lead to a critical overall risk of bias. Additionally, inconsistencies in follow-up methods and the potential for selective reporting in the meta-analysis contribute to the overall risk of bias. |
| **Lau 2014** | **Moderate** | **Moderate** | **Low** | **Low** | **Moderate** | **Low** | **Moderate** | **Moderate** |
| **Rationale for judgement** | While the study controlled for many factors, such as age, tumor stage, and grade, there may still be residual confounding from other unmeasured factors, like fertility preferences, hormonal levels, or underlying comorbidities. | Patients who expressed a desire for ovarian preservation may differ systematically from those who underwent oophorectomy (e.g., younger age, better health). Additionally, some patients had ovarian preservation due to incidental findings, which could introduce selection bias. | The classification of interventions was straightforward and based on surgical records, minimizing the risk of misclassification. | The study did not report any deviations from the intended interventions, and no active changes in ovarian preservation or oophorectomy were made post-surgery. | The completeness of the follow-up data is not clearly reported, particularly in terms of patients lost to follow-up or the number of censored patients in the survival analysis. | Outcomes such as disease-free survival and overall survival are standard and objectively measured using medical records, pathology reports, and follow-up data. | The study does not mention a pre-specified protocol, and the choice of outcomes could be influenced by the data available in the retrospective review. | The study faces several challenges due to its retrospective nature, including potential confounding, selection bias, and incomplete follow-up. |
| **Gonthier 2016** | **Critical** | **Moderate** | **Low** | **Moderate** | **Moderate** | **Low** | **Moderate** | **Critical** |
| **Rationale for judgement** | Only reported patients with grade 2 or 3 endometrial cancer. In addition, the patients' age, race, and other demographic factors were significantly different between groups, and although these factors were adjusted for in logistic regression and Cox models, other potential confounders such as comorbidities, fertility preferences, or socio-economic status were not fully accounted for. | The retrospective selection of patients and the fact that some treatments (like ovarian preservation) were less common during certain periods may introduce bias. | The classification of interventions appears to be clearly and consistently applied, minimizing the risk of bias from intervention misclassification. | Differences in post-treatment care or follow-up might have introduced bias, particularly since uterine and ovarian preservation is relatively recent, and the clinical care pathways for these patients could vary. | The study excluded 112 patients due to missing data on management type. However, there is no detailed reporting of missing follow-up data or survival outcomes, particularly for the conservative management groups. | Outcome measures are objective (e.g., time to death, survival), and the SEER database is a reliable source for such data, leading to a low risk of bias in outcome measurement. | The lack of a pre-specified protocol and potential for selective reporting of favorable results or significant comparisons introduces a moderate risk of bias in the selection of reported outcomes. | The main sources of bias include potential confounding, missing data, and selection of participants into the study, despite efforts to adjust for certain variables in the analysis. |
| **Matsuo 2016** | **Moderate** | **Low** | **Low** | **Moderate** | **Moderate** | **Low** | **Low** | **Moderate** |
| **Rationale for judgement** | While many confounders were accounted for, the use of SEER data (which lacks clinical details like comorbidities or hormonal status) may lead to some residual confounding. | Although some cases were excluded due to missing data, the population-based nature of SEER data minimizes significant selection bias. | The classification of patients into ovarian conservation versus oophorectomy groups was based on surgical codes in the SEER database, which should be reliable. | Differences in post-surgical treatment between the groups could introduce bias, though this is somewhat mitigated by adjusting for treatment patterns in the analysis. | The study excluded cases with unknown oophorectomy status, which might introduce bias if these cases were not missing at random. | The outcomes (overall survival and cause-specific survival) were measured using SEER’s robust cancer-specific survival and vital status tracking, which is reliable. However, SEER's cause-of-death data may not be as precise as clinically adjudicated death causes, especially when differentiating cardiovascular deaths from other chronic disease deaths. | There is no evidence of selective reporting of outcomes, and the main outcomes are transparently presented. | Some risk of bias primarily due to potential unmeasured confounders, and missing data on certain clinical factors. |
| **Wright 2016** | **Moderate** | **Low** | **Low** | **Low** | **Moderate** | **Low** | **Low** | **Moderate** |
| **Rationale for judgement** | Despite adjustments, unknown or unmeasured confounders may still exist, particularly regarding reasons for ovarian conservation vs. BSO. | The use of a nationwide, comprehensive registry with broad inclusion criteria minimizes bias in participant selection. | The interventions (ovarian conservation vs. BSO) were classified based on surgical procedure records in the database, which are likely to be accurate. | Given that the data were derived from retrospective records and the interventions were clearly defined and executed, the risk of deviation from intended interventions is minimal. | The study excludes patients with incomplete data on certain variables, but it does not explicitly mention the extent of missing data or how this issue was handled (e.g., through imputation). | The primary outcome of interest (overall survival) was measured using death records, which are a reliable source of outcome data. | No evidence suggests selective reporting, and the results appear comprehensive and aligned with the pre-defined objectives. | The study is well-designed for a retrospective analysis, with large sample size and robust statistical adjustments for confounding factors. However, there is a moderate risk of confounding, and the study does not address why certain patients or clinicians chose ovarian conservation versus oophorectomy, which could influence the results. |
| **Hou 2017** | **Moderate** | **Low** | **Low** | **Moderate** | **Moderate** | **Low** | **Low** | **Moderate** |
| **Rationale for judgement** | While propensity score matching reduces bias, the lack of detailed clinical factors such as hormonal therapy usage may still introduce residual confounding. | The population-based nature of SEER data minimizes significant bias. | The classification of interventions (ovarian preservation VS BSO) relied on SEER’s surgical coding system, which is generally reliable. However, misclassification of oophorectomy status may occur if coding errors exist. | Differences in post-surgical treatment between the groups could introduce bias, though this is somewhat mitigated by adjusting for treatment patterns in the analysis. | The study handles missing data through exclusions, but missing clinical information from SEER (such as hormonal therapy use) limits the robustness of the conclusions. | The study’s outcomes (overall and cancer-specific survival) were measured using SEER’s tracking system, which is robust. However, SEER’s cause-specific survival data may lack the precision of clinical adjudication, particularly in distinguishing cancer-related deaths from other causes. | The study appears to report all relevant results without selective reporting. | The study uses rigorous methods, however, there are some limitations, such as potential residual confounding from unmeasured variables and missing clinical data, that contribute to a moderate overall risk of bias. |
| **Matsuo 2019** | **Moderate** | **Low** | **Low** | **Moderate** | **Low** | **Low** | **Low** | **Moderate** |
| **Rationale for judgement** | The study accounts for some important confounders like age, hormonal therapy, and surgical factors. However, there is limited adjustment for other relevant confounders such as BMI, comorbidities, and tumor characteristics in the composite outcome. | Women were selected from institutional databases from 23 centers across the US and Japan, ensuring comprehensive coverage of eligible participants. Exclusion criteria were also appropriate. There is no indication of differential selection that could lead to bias. | The classification of ovarian conservation (retention of at least one ovary) and oophorectomy (absence of ovaries) was clearly defined based on surgical records, reducing the risk of misclassification. | There is no mention of whether patients received post-hysterectomy therapy. Additionally, the use of ERT could introduce variability in outcomes, as some patients who underwent oophorectomy were given ERT, but this effect is not completely addressed. | The study does not indicate significant loss to follow-up, and survival data appear to be available for the majority of patients. | Disease-free survival (DFS) and overall survival (OS) were clearly defined, and standard survival analysis methods were used. | The study reports both primary and secondary outcomes as predefined in the study objectives. There is no evidence of selective reporting of outcomes. | The study’s design and execution appear sound, but there are some limitations concerning unadjusted confounders and potential deviations from interventions. |
| **Shin 2020** | **Moderate** | **Low** | **Low** | **Moderate** | **Low** | **Low** | **Low** | **Moderate** |
| **Rationale for judgement** | The ovarian preservation group had a significantly earlier stage of cancer and younger patients, indicating potential confounding. Although multivariate Cox regression was performed, other potential confounders, like comorbidities or hormonal status, were not accounted for, which could bias the results. | There is no indication of differential selection that would systematically favor one intervention group over the other. | The classification of interventions (ovarian preservation VS BSO) was based on surgical records, which are likely reliable and accurate. There is no indication of misclassification of the intervention groups, reducing the risk of bias in this domain. | The imbalance in FIGO stage and age between the two groups indicates that patient characteristics may have influenced the decision for ovarian preservation or removal, introducing bias. | The study does not mention any significant loss to follow-up, and the five-year overall survival and recurrence-free survival rates were calculated for nearly all included patients. | Outcome measures, including overall survival and recurrence-free survival, are objectively defined and are standard endpoints in oncologic studies. The use of medical records and Kaplan-Meier survival curves for analysis suggests a reliable measurement of outcomes across patients. | The study appears to report all relevant results, including both overall survival and recurrence-free survival outcomes, without selective reporting. | The study has a moderate overall risk of bias, primarily due to confounding from differences in patient characteristics (age and FIGO stage) between the ovarian preservation and removal groups. |
| **Akgor 2022** | **Moderate** | **Moderate** | **Low** | **Moderate** | **Low** | **Low** | **Low** | **Moderate** |
| **Rationale for judgement** | The study adjusted for several variables like tumor grade, size, and lymphovascular invasion in multivariate analysis. However, certain factors like the significantly higher ratio of patients under 30 years old in the ovarian preservation surgery group (20.4% vs. 9.6%, P = 0.021) and differences in tumor size (>2 cm was more frequent in the BSO group) may indicate residual confounding. | The exclusion of patients aged >40 years and those with advanced-stage disease may have introduced some bias, particularly as the chosen age threshold (≤40 years) was arbitrary. Additionally, while patients were informed about the risks and benefits of oophorectomy, the decision to preserve ovaries or perform a BSO was left to clinicians, which may lead to potential selection bias based on patient characteristics and clinical preferences. | The intervention (ovarian preservation VS BSO) was based on clear surgical decisions and medical records, which are unlikely to have been misclassified. | Although the surgical decisions followed international guidelines, deviations from intended interventions may have occurred due to differences in individual centers’ protocols and clinician decisions. Additionally, the study spans over several years (2007-2019), during which treatment protocols may have changed, potentially influencing the outcome of ovarian preservation or removal, thus introducing bias. | There is no mention of significant missing data in the study. Data validation and quality checks were conducted with the participating centers, reducing the likelihood of bias due to missing data. | The outcomes, including overall survival and recurrence-free survival, are standard and objective measures in oncology research. The study used established methods such as the Kaplan-Meier survival analysis and Cox proportional hazard models to assess outcomes, and there is no indication that the outcomes were differentially assessed between intervention groups. | The study reports both overall survival and recurrence-free survival for the ovarian preservation and BSO groups, as well as the results of univariate and multivariate analyses, without evidence of selective reporting. | Potential confounding factors, such as differences in age and tumor size between the ovarian preservation and BSO groups, and possible deviations from intended interventions due to variations in clinical practice across centers. |
| **Nasioudis 2022** | **Critical** | **Low** | **Low** | **Moderate** | **Moderate** | **Low** | **Low** | **Critical** |
| **Rationale for judgement** | Only reported patients with grade 2 or 3 endometrial cancer. In addition, the study attempted to control for key confounding variables like insurance, comorbidities, performance of lymphadenectomy, disease substage, tumor size, and grade using a Cox model. However, patients who had ovarian preservation were less likely to undergo lymphadenectomy (52% vs. 76.2%, p<0.001), which could confound survival outcomes since lymphadenectomy is an important staging and treatment procedure in endometrial cancer. | The inclusion and exclusion criteria were clearly defined based on data from the National Cancer Database (NCDB). The patient cohort was selected based on specific clinical characteristics (e.g., age ≤45 years, FIGO stage I, and tumor grades 2 or 3). | The intervention (ovarian preservation or BSO) was classified based on detailed surgery codes in the NCDB. There is no indication of misclassification of interventions, as ovarian preservation and BSO were clearly defined and extracted from the database. | The decision to perform ovarian preservation was likely influenced by patient age and clinician preference, which could have introduced deviations from standardized treatment protocols. Additionally, the temporal trend showing a decrease in ovarian preservation suggests that treatment decisions may have evolved over time, potentially impacting outcomes. | The study mentioned that key variables like peritoneal cytology and lymph-vascular invasion were missing for a significant proportion of cases (data introduced after 2010). While the missing data were not included in survival analyses, their absence could limit the accuracy of conclusions regarding risk factors like lymph-vascular invasion and metastasis. The exclusion of patients with missing data may introduce bias. | The primary outcome was overall survival, which is an objective measure. Survival data were derived from the NCDB, and Kaplan-Meier survival curves were used, a standard method in cancer outcome studies. | The study comprehensively reports both the overall survival and multivariate Cox regression models controlling for confounding factors. It also provides detailed analyses for different subgroups (e.g., tumor grade, depth of myometrial invasion). There is no indication of selective reporting or suppression of unfavorable results. | Although there were efforts to adjust for key confounding factors, including only grade 2 or 3 endometrial cancer and the significant difference in lymphadenectomy rates between the ovarian preservation and BSO groups poses a risk of residual confounding. Additionally, the missing data on peritoneal cytology and lymph-vascular invasion for a large subset of patients introduces some uncertainty. |

**BSO:** Bilateral Salpingo-Oophorectomy

**ERT:** Estrogen Replacement Therapy
