# Supplementary material for: Impact of ovarian preservation on survival for premenopausal women with early‐stage endometrial carcinoma: A systematic review and meta‐analysis
Source: Int J Gynaecol Obstet. 2026 Jun 11;175(2):608–18. doi: 10.1002/ijgo.71101 (PMC13674844; doi:10.1002/ijgo.71101)
Supplement: Supplementary file 2 [file IJGO-175-608-s005.docx]

**Supplementary Material 2.** Central search strategy.

#1	MeSH descriptor: [Endometrial Neoplasms]

#2	(endometr* near/5 (cancer* or tumor* or tumour* or neoplas* or malignan* or carcinom* or adenocarcinom*))

#3	(uter* and lining and (cancer* or tumor* or tumour* or neoplas* or malignan* or carcinom* or adenocarcinom*))

#4	#1 or #2 or #3

#5	(ovar* near/5 (sparing* or $preserv* or conserv* or protect*))

#6	*Organ Sparing Treatment*

#7	MeSH descriptor: [Ovariectomy] this term only

#8	$oophor* or ovariectomy* or annessiectomy* or BSO

#9	(ovar* near/5 (biopsy or remov* or excision*))

#10	#5 or #6 or #7 or #8 or #9

#11	#4 and #10
