# Supplementary material for: Impact of ovarian preservation on survival for premenopausal women with early‐stage endometrial carcinoma: A systematic review and meta‐analysis
Source: Int J Gynaecol Obstet. 2026 Jun 11;175(2):608–18. doi: 10.1002/ijgo.71101 (PMC13674844; doi:10.1002/ijgo.71101)
Supplement: Supplementary file 4 [file IJGO-175-608-s011.docx]

**Supplementary Material 3.** Embase (Ovid) search strategy.

1.	exp Endometrial Neoplasms/

2.	(endometr* adj5 (cancer* or tumor* or tumour* or neoplas* or malignan* or carcinom* or adenocarcinom*))

3.	(uter* and lining and (cancer* or tumor* or tumour* or neoplas* or malignan* or carcinom* or adenocarcinom*))

4.	1 or 2 or 3

5.	(ovar* adj5 (sparing* or preserv* or conserv* or protect*))

6.	*Organ Sparing Treatments

7.	Ovariectomy/

8.	(oophor* or ovariectomy* or annessiectomy* or BSO)

9.	(ovar* adj5 (biopsy or remov* or excision*))

10.	5 or 6 or 7 or 8 or 9

11.	4 and 10

12.	crossover procedure/

13.	double-blind procedure/

14.	randomized controlled trial/

15.	single-blind procedure/

16.	random*

17.	factorial*

18.	(crossover* or cross over* or cross-over*)

19.	placebo*

20.	(double* adj blind*)

21.	(singl* adj blind*)

22.	assign*

23.	allocat*

24.	volunteer*

25.	Clinical study/

26.	Case-Control Studies/

27.	Longitudinal study/

28.	Retrospective study/

29.	Prospective study/

30.	Cohort analysis/

31.	(Cohort adj (study or studies))

32.	(Case control adj (study or studies))

33.	(follow up adj (study or studies))

34.	(observational adj (study or studies))

35.	(cross sectional adj (study or studies))

36.	12 or 13 or 14 or 15 or 16 or 17 or 18 or 19 or 20 or 21 or 22 or 23 or 24 or 25 or 26 or 27 or 28 or 29 or 30 or 31 or 32 or 33 or 34 or 35

37.	11 and 36
