# Supplementary material for: Impact of ovarian preservation on survival for premenopausal women with early‐stage endometrial carcinoma: A systematic review and meta‐analysis
Source: Int J Gynaecol Obstet. 2026 Jun 11;175(2):608–18. doi: 10.1002/ijgo.71101 (PMC13674844; doi:10.1002/ijgo.71101)
Supplement: Supplementary file 5 [file IJGO-175-608-s012.docx]

**Supplementary Material 1.** MEDLINE search strategy.

(endometr* OR uter*) AND (cancer OR tumor OR tumour OR neoplas* OR malignan* OR carcinom* OR adenocarcinom*) AND (ovar* OR fertil* OR adnex*) AND (sparing OR preserv* OR protect) AND (oophorect* OR ovariect* OR adnexect* OR BSO OR remov* OR biops* OR excision)
