# Supplementary material for: Impact of ovarian preservation on survival for premenopausal women with early‐stage endometrial carcinoma: A systematic review and meta‐analysis
Source: Int J Gynaecol Obstet. 2026 Jun 11;175(2):608–18. doi: 10.1002/ijgo.71101 (PMC13674844; doi:10.1002/ijgo.71101)
Supplement: Supplementary file 6 [file IJGO-175-608-s013.docx]

**Supplementary Table 1.** Criteria for risk of bias judgements according to ROBINS-I tool.

| **JUDGEMENT** | **BIAS DUE TO CONFOUNDING** | **BIAS IN SELECTION OF PARTICIPANTS INTO THE STUDY** | **BIAS IN CLASSIFICATION OF INTERVENTIONS** | **BIAS DUE TO DEVIATIONS FROM THE INTENDED INTERVENTION** | **BIAS DUE TO MISSING DATA** | **BIAS IN MEASUREMENT OF OUTCOMES** | **BIAS IN SELECTION OF THE REPORTED RESULT** |
| --- | --- | --- | --- | --- | --- | --- | --- |
| **LOW RISK OF BIAS (THE STUDY IS COMPARABLE TO A WELL‐PERFORMED RCT WITH REGARD TO THIS DOMAIN)** | No confounding expected. | All participants who would have been eligible for the target trial were included in the study *and* start of follow‐up and start of intervention coincide for all participants. | Intervention status is well‐defined and based solely on information collected at the time of intervention. | No bias due to deviation from the intended intervention is expected, e.g. if both the intervention and comparator are implemented over a short time period, and subsequent interventions are part of routine medical care, or if the specified comparison relates to initiation of intervention regardless of whether it is continued. | Data were reasonably complete; *or* proportions of and reasons for missing participants were similar across intervention groups; *or* analyses that addressed missing data are likely to have removed any risk of bias. | The methods of outcome assessment were comparable across intervention groups; *and* the outcome measure was unlikely to be influenced by knowledge of the intervention received by study participants (i.e. is objective) *or*the outcome assessors were unaware of the intervention received by study participants; *and* any error in measuring the outcome is unrelated to intervention status. | There is clear evidence (usually through examination of a pre‐registered protocol or statistical analysis plan) that all reported results correspond to all intended outcomes, analyses, and subcohorts. |
| **MODERATE RISK OF BIAS (THE STUDY IS SOUND FOR AN NRCT WITH REGARD TO THIS DOMAIN BUT CANNOT BE CONSIDERED COMPARABLE TO A WELL‐PERFORMED RCT)** | Confounding expected, all known important confounding domains appropriately measured and controlled for; *and* reliability and validity of measurement of important domains were sufficient, such that we do not expect serious residual confounding. | Selection into the study may have been related to intervention and outcome, but the authors used appropriate methods to adjust for the selection bias; *or* start of follow‐up and start of intervention do not coincide for all participants, but (a) the proportion of participants for which this was the case was too low to induce important bias; (b) the authors used appropriate methods to adjust for the selection bias; or (c) the review authors are confident that the rate (hazard) ratio for the effect of intervention remains constant over time. | Intervention status is well‐defined, but some aspects of the assignments of intervention status were determined retrospectively. | Bias due to deviation from the intended intervention is expected, and switches, co‐interventions, and some problems with intervention fidelity are appropriately measured and adjusted for in the analyses. Alternatively, most (but not all) deviations from intended intervention reflect the natural course of events after initiation of intervention. | Proportions of missing participants differ across interventions; *or* reasons for missingness differ minimally across interventions; *and* missing data were not addressed in the analysis. | The methods of outcome assessment were comparable across intervention groups; *and* the outcome measure is only minimally influenced by knowledge of the intervention received by study participants; *and* any error in measuring the outcome is only minimally related to intervention status. | The outcome measurements and analyses are consistent with an a priori plan; or are clearly defined and both internally and externally consistent; *and* there is no indication of selection of the reported analysis from among multiple analyses; *and* there is no indication of selection of the cohort or subgroups for analysis and reporting on the basis of the results. |
| **SERIOUS RISK OF BIAS (THE STUDY HAS SOME IMPORTANT PROBLEMS)** | Switches in treatment, co‐interventions, or problems with implementation fidelity are apparent and are not adjusted for in the analyses. | Proportions of missing participants differ substantially across interventions; *or* reasons for missingness differ substantially across interventions; *and* missing data were addressed inappropriately in the analysis; *or* the nature of the missing data means that the risk of bias cannot be removed through appropriate analysis. | The methods of outcome assessment were not comparable across intervention groups; *or* the outcome measure was subjective (i.e. likely to be influenced by knowledge of the intervention received by study participants) and was assessed by outcome assessors aware of the intervention received by study participants; *or* error in measuring the outcome was related to intervention status. | Switches in treatment, co‐interventions, or problems with implementation fidelity are apparent and are not adjusted for in the analyses. | Proportions of missing participants differ substantially across interventions; *or* reasons for missingness differ substantially across interventions; *and* missing data were addressed inappropriately in the analysis; *or* the nature of the missing data means that the risk of bias cannot be removed through appropriate analysis. | The methods of outcome assessment were not comparable across intervention groups; *or* the outcome measure was subjective (i.e. likely to be influenced by knowledge of the intervention received by study participants) and was assessed by outcome assessors aware of the intervention received by study participants; *or* error in measuring the outcome was related to intervention status. | Outcome measurements or analyses are internally or externally inconsistent; *or* there is a high risk of selective reporting from among multiple analyses; *or* the cohort or subgroup is selected from a larger study for analysis and appears to be reported on the basis of the results. |
| **CRITICAL RISK OF BIAS (THE STUDY IS TOO PROBLEMATIC TO PROVIDE ANY USEFUL EVIDENCE ON THE EFFECTS OF THE INTERVENTION)** | Substantial deviations from the intended intervention are present and are not adjusted for in the analysis. | (Unusual) There were critical differences between interventions in participants with missing data that were not, or could not, be addressed through appropriate analysis. | The methods of outcome assessment were so different that they cannot reasonably be compared across intervention groups. | Substantial deviations from the intended intervention are present and are not adjusted for in the analysis. | (Unusual) There were critical differences between interventions in participants with missing data that were not, or could not, be addressed through appropriate analysis. | The methods of outcome assessment were so different that they cannot reasonably be compared across intervention groups. | There is evidence or strong suspicion of selective reporting of results, and the unreported results are likely to be substantially different from the reported results. |
| **NO INFORMATION ON WHICH TO BASE A JUDGEMENT ABOUT RISK OF BIAS FOR THIS DOMAIN** | No information is reported on whether there is deviation from the intended intervention. | No information is reported about missing data or the potential for data to be missing. | No information is reported about the methods of outcome assessment. | No information is reported on whether there is deviation from the intended intervention. | No information is reported about missing data or the potential for data to be missing. | No information is reported about the methods of outcome assessment. | There is too little information to make a judgement (e.g. if only an abstract is available for the study). |
